# Potential and optimization of two-phase anaerobic digestion of oil refinery waste activated sludge and microbial community study

**DOI:** 10.1038/srep38245

**Published:** 2016-12-01

**Authors:** Qinghong Wang, Ying Liang, Peng Zhao, Qing X. Li, Shaohui Guo, Chunmao Chen

**Affiliations:** 1 State Key Laboratory of Heavy Oil Processing, Beijing Key Laboratory of Oil & Gas Pollution Control, China University of Petroleum, Beijing 102249, PR China; 2 State Key Laboratory of Mycology, Institute of Microbiology, Chinese Academy of Sciences, Beijing 100101, PR China; 3 Department of Molecular Biosciences and Bioengineering, University of Hawaii at Manoa, Honolulu, HI 96822, USA

## Abstract

Oil refinery waste activated sludge produced from oil wastewater biological treatment is a major industrial sludge. Two-phase anaerobic digestion of oil refinery waste activated sludge was studied for the first time. Thermal pretreatment under 170 °C is effective on sludge solubilization. At the optimum hydrolytic-acidogenic condition which was pH of 6.5, temperature of 55 °C and HRT of 2 days, 2754 mg/L volatile fatty acids (VFAs) were produced and acetic acid and butyric acid were the key components. Comparative studies of single-phase and two-phase anaerobic digestion in terms of organic removal, biogas production and methane concentration were conducted. The cumulative methane production and soluble COD (SCOD) removal efficiency in the two-phase system were 228 mL/g COD added and 77.8%, respectively, which were 1.6 and 2.1 times higher than those in single-phase anaerobic digestion. Such improved performance is attributed to intensification of dominant microbial population in separated reactors. *Caloramator, Ureibacillus, Dechloromonas, Petrobacter*, and *T78* played important roles in hydrolytic-acidification and oil-organics degradation. Syntrophic bacteria in the family Porphyromonadaceae and the genus *Anaerobranca* provide acetate for methanogen. The results demonstrated the potential and operating condition of two-phase anaerobic digestion in treatment of oil refinery waste activated sludge.

Oil refinery waste activated sludge, which is produced in oil wastewater biological treatment process, is a major industrial sludge. It contains pathogens and toxic inorganic and persistent organic pollutants^1^,^2^. Efficient and effective handling and disposal of oil refinery waste activated sludge can minimize risks to the environment and human health. In addition, oil compounds present in such sludge contain high calorific values of 17000–19000 kJ/kg^2^. It therefore is a valuable energy resource. In this sense, pyrolysis and incineration have been deemed as effective methods to destroy organics into fuel, pyrolytic gas or carbon dioxide, and heat recovery^3^,^4^. But high dehydration cost and heat-resistant equipment restrict their utilization in large scale. Some studies have been performed moderate condition treatment of refinery sludge, such as land farming with aerobic microbes to convert hydrocarbons into CO_2_^5^. However, a large surface area is needed and air/groundwater pollution problems could be caused. Alternatively, anaerobic digestion is an economic and effective technique for sludge stabilization, utilization and reclamation^6^. Through this process, waste can be treated and methane-containing biogas is released. Anaerobic digestion is common in municipal waste activated sludge treatment and is increasingly applied in the industrial wastes stabilization^7^. However, there were few studies on anaerobic digestion of refinery waste activated sludge. The potential and optimum running conditions still need to be investigated.

Efficient treatment of refinery sludge in conventional single-phase anaerobic digestion could be challenging due to complicated matrix present in such sludge, including oil and refractory organics. Hydrolysis, acidogenesis, and methanogenesis all take place in one pot during a single-phase anaerobic digestion process. The system requires a long retention time and neutral pH because methanogens are slow-growing and condition-sensitive. A condition favorable for methanogenesis may suppress hydrolytic-acidogenic bacteria^8^. Two-phase anaerobic digestion consists hydrolytic-acidogenic and methanogenesis stages, which shows better stability, shorter retention time and greater methane production rate than conventional single-phase anaerobic digestion. The phase separation can improve the performance of hydrolytic-acidogenic stage and is appropriate to complex and high-strength solid wastes^9^. A comparative study on single-phase and two-phase anaerobic digestion of refinery waste activated sludge should be conducted to verify the above statement.

Pretreatments are often used in anaerobic digestion of sludge, which can accelerate the hydrolysis of biomass and improve biodegradability of sludge^10^. After effective pretreatments, these substances in the liquid or colloidal state are, therefore, accessible to anaerobes. Pretreatment methods vary from chemical processes (such as acid, alkaline pretreatment), physical processes (such as thermal, ultrasonic pretreatment) to their combinations. The performance of these different methods varied, and their hydrolysis efficiency relied mainly on sludge samples. To select feasible pretreatment method for refinery wastes activated sludge, comparative experiments are needed.

The hydrolytic-acidogenic conditions also need to be optimized according to waste characteristics^11^. Although, the specific operational conditions in anaerobic digestion have been extensively studied, relevant literature on hydrolytic-acidogenic stage is still scare. Moreover, there is a lack of knowledge about running conditions of hydrolytic-acidogenic reactor fed with refinery waste activated sludge. It is well known that operational conditions affect the bacterial community and predominant acidogens^12^. Temperature, HRT and pH are important parameters manipulating hydrolytic-acidogenic efficiencies and products. Selective enrichment of oil-decomposing acidogens is important for degradation of oil hydrocarbons in refinery sludge. Therefore, operation conditions of the hydrolytic-acidogenic stage for the two-phase anaerobic digestion of refinery waste activated sludge need to be optimized.

In the present study, a two-phase anaerobic digestion was studied for the treatment of oil refinery waste activated sludge in comparison with single phase anaerobic digestion. Different pretreatment methods were compared for the effectiveness, which included thermal, alkali, ultrasonic and ultrasonic-thermal pretreatments. Operational temperature, pH and HRT were optimized for effective hydrolytic-acidogenosis of pretreated sludge. Methane production, organic removal and microbial communities in both single-phase and two-phase anaerobic digestion were compared.

## Results

### Effect of pretreatment methods on sludge disintegration

**Figure 1:**
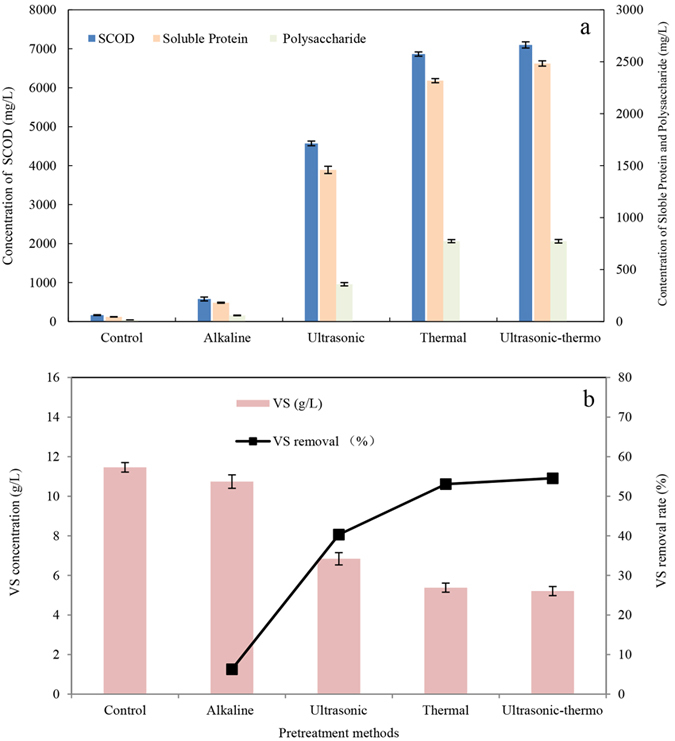
The effectiveness of different pretreatments on solubilisation of sludge, focus on SCOD, protein and polysaccharide concentrations (**a**) and SS removal (**b**).

The effects of ultrasonic, thermal, alkali and ultrasonic-thermal pretreatments on sludge solubilisation and VS reduction were investigated (Fig. 1). From Fig. 1a, the concentration of SCOD, soluble protein and polysaccharide in thermal treated sludge was 6865 mg/L, 2318 mg/L and 773 mg/L, respectively, which were almost 1.25–1.5 fold higher than those in ultrasonic treated sludge and 6–6.5 times higher than those in alkaline treated sludge. The concentration of SCOD, soluble protein and polysaccharide in ultrasonic-thermal treated sludge was 7099 mg/L, 2483.65 mg/L and 773.32 mg/L, respectively. Figure 1b shows the influence of different pretreatments on VS removal. The removal efficiency by thermal, ultrasonic-thermal, ultrasonic and alkaline treatments were 53.10%, 54.53%, 40.3% and 6.3%, respectively.

### Optimization of the hydrolytic-acidogenic phase

**Figure 2:**
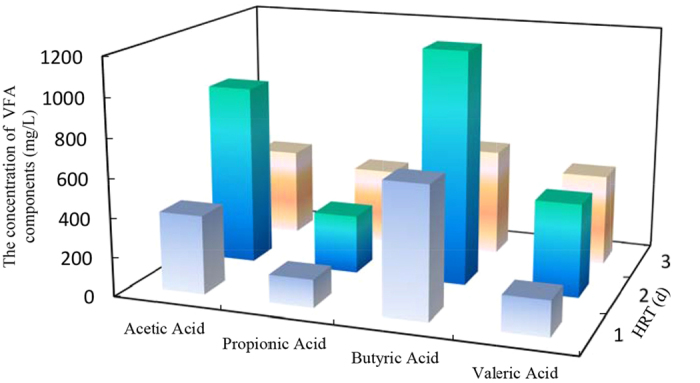
The concentration of individual VFAs at pH of 6.5 and temperature of 55 °C.

Table 1 presented the effects of temperature and pH on VFA production at each experimental HRTs. The highest VFA concentration was 1801.5 ± 44.4 mg/L at 55 °C and HRT of 2 days. In mesophilic digestion, the highest value was 486.4 ± 17.2 mg/L at HRT of 4 days. Under each HRTs, VFA production was higher at thermophilic condition than that at mesophilic circumstance. Besides, VFA production was considerably affected by pH. The optimum pH was ranged from 6.5 to 7.0, and the maximum VFA concentration of 2754.0 ± 25.5 mg/L achieved at pH of 6.5 and HRT of 2 days. With the reduction of pH from 6.5 to 5.0, VFA production decreased. Moreover, VFA production increased at the first 1–2 days and then decreased under each pH conditions. Furthermore, to investigate the variation of VFA components with hydrolytic-acidogenic time, three samples were detected at HRT of 1, 2, and 3 days, shown in Fig. 2. Butyric acid and acetic acids were the dominating components at each HRT but the relative content was different. The maximum value of butyric acid and acetic acid were 1193.8 mg/L and 926.7 mg/L at HRT of 2 days. Both of them accounted for over 70% of total VFA. When HRT prolonged to 3 days, the concentration of acetic acids and butyric acids decreased but propionic and valeric acids increased.

### Two-phase vs. single-phase anaerobic digestion

**Figure 3:**
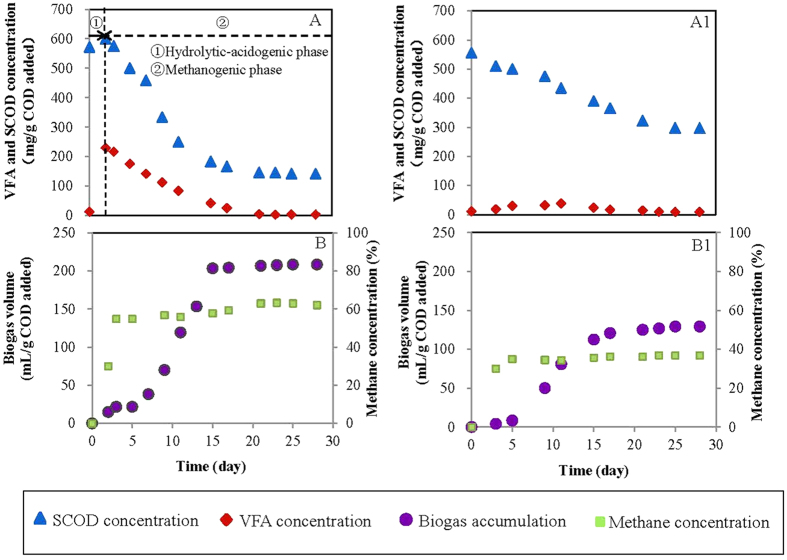
The comparison of organic removal and methane production in two systems (**A**) VFA & SCOD concentration in two-phase anaerobic digestion system (**B**) biogas production & methane concentration in two-phase anaerobic digestion system; (A1) VFA & SCOD concentration in single-phase anaerobic digestion (B1) biogas production & methane concentration in single-phase anaerobic digestion.

The comparative results of organics removal and methane production between two phase (hydrolytic-acidogenic + methanogensis phase) and single phase anaerobic digestion during 15 days were shown in Fig. 3. Initial SCOD and VFA concentration in the two systems were same. Through hydrolytic-acidogenic treatment, SCOD increased to 655 mg/g COD added and VFA of 250 mg/g COD added was produced (Fig. 3A). These soluble organics were quickly utilized in the methanogenic phase during 15 days, and SCOD removal rate reached up to 77.8%. Meanwhile, biogas was rapidly produced when feeding with hydrolytic-acidogenic sludge and accumulated to 228 mL/g COD added at the 15^th^ day (Fig. 3B). Methane concentration increased to 54.8% within 5 days and then fluctuated around 60%. In contrast, single-phase anaerobic digestion showed low methane production rate and poor organic removal efficiency (Fig. 3A1, B1). Concerning the single-phase system, the thermal-pretreated sludge with initial SCOD of 572 mg/g COD added was digested, only 48.1% SCOD was removed to the end of treatment. VFA concentration was always below 40 mg/g COD added in the system. From 5^th^ day, biogas was gradually produced, and the accumulative biogas volume was 129 mL/g COD added which was almost 40% lower than that from two-phase system. Besides, methane concentration was less than 40% during the whole period of single-phase anaerobic digestion.

### Oil conversion in the two-phase system

**Figure 4:**
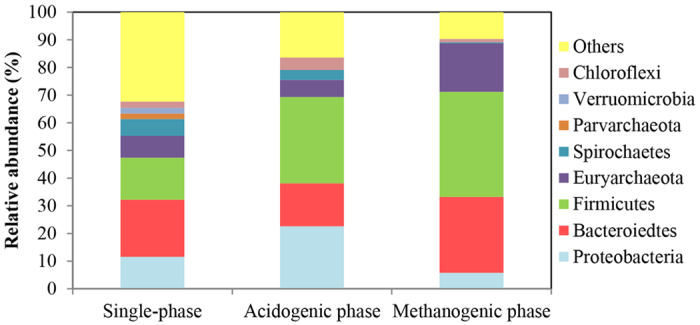
Gas chromatography-mass spectrometry results of oil products before (**A**) and after (**B**) two-phase anaerobic digestion: 16.27 dodecane; 21.09 tetradecane; 22.87 pentadecane; 25.08 2,6,10-trimethylpentadecane; 25.81 pristane; 27.05 Adenosine; 27.87 dibutyl phthalate; 28.87 10,13-eicosadienoic acid, methyl eater; 30.00 octadecane; 33.96 1,2-benzenedicarboxylic acid, diisooctyl ester; 35.43 5α-cholestane; 38.05 lanostane; 40.13 Hopane; 40.64 ambreinolide; 42.38 acetic acid, 4-acetoxy-3-(3,7,11-trimethyi-dodeca-2,6,10-trienyl)-phenyl ester; 43.96 Lupan-3-one; 46.06 3,12-Oleandione.

Oil conversion in the two-phase anaerobic digestion was studied. Oil content of the refinery waste activated sludge was 5.3% g/g-TS. After two-phase anaerobic digestion, 1.2% g/g-TS of oil were left in the sludge residue. The oil components before and after two-phase anaerobic digestion were subjected to GC-MS, shown in Fig. 4. A total of 68 different chemicals were detected, and these chemicals belong to 9 major groups in the initial sample, including alkenes, alcohols, ketones, heteroatom-containing compounds, esters, aromatics, organic acids, aldehydes, and phenols. Among them, the relative contents of alkenes had high proportion (76.53%), with carbon number ranged from C12 to C30. Alcohols, ketones, heteroatom-containing compounds, and esters were followed which accounted for 7.35%, 5.56%, 5.43%, and 3.14%, respectively. In the sludge residue, 64 different compounds were detected belonging to above-mentioned groups. The main components were alkenes, esters and alcohols accounted for 46.82%, 20.49% and 19.38%, respectively.

### Microbial community

**Figure 5:**
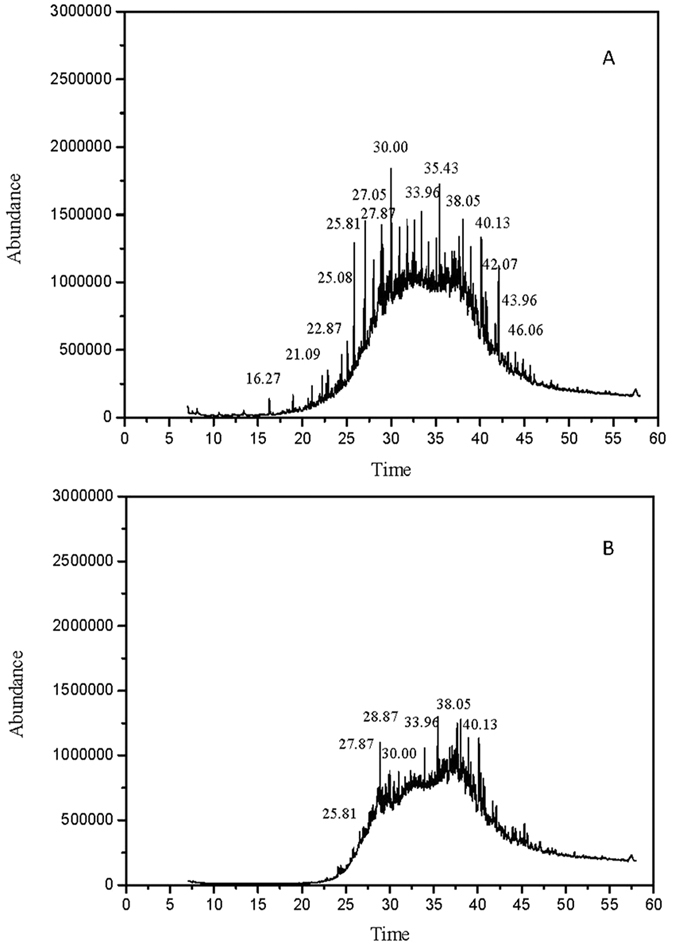
Taxonomic classification of archaeal and bacterial phylum in the single-phase, hydrolytic-acidogeni and methanogenic reactors. Minor taxons with relative abundance of <1% in any sample were grouped together as others.

A total of 264938 sequences were obtained which were clustered to calculate operational taxonomic units (OTUs) using 97% identity as a cutoff, resulting in 11679 OTUs. The species richness and taxonomic diversity expressed in Chao1 and Shannon index were calculated by QIIME software. The Chao1 (species richness) index was 9030, 8205 and 5909, while the Shannon (taxonomic diversity) index were 10.4, 9.4, and 8.6 among samples in the single-phase, hydrolytic-acidogenic and methanogenic reactors, respectively. Figure 5 showed the relative abundance of archaeal and bacterial phylum in three reactors. It can be seen that Bacteroidetes, Firmicutes and Protebacteria were the most abundant phyla, which were over 60% of the total relative abundance in two-phase systems and over 40% in single-phase reactor. The detailed bacterial and archaea populations were shown in Table 2. The relative abundance of archaeal genera and bacterial at family/genus level in different reactors were listed. All detected Archaea belonged to class Methanomicrobia of the phylum Euryarchaeota, which were known as methanogenic microorganisms. The relative abundances of Methanomicrobia in the single phase, acidogenic and methanogenic reactors were 8.1%, 6.2% and 17.6%, respectively. 13 genus/family of bacteria, belonging to 8 classes, were detected with relative abundance above 1%. Genes of *Caloramator* (15.1%), *Ureibacillus* (5.3%), *Dechloromonas* (2.7%), *Petrobacter* (4.3%), and *T78* (4.5%) were richer in hydrolytic-acidogenic reactor comparing with those in two others. The relative abundance of *Porphyromonadaceae* (24.5%) and *Anaerobranca* (6.1%) in methanogenic phase was higher in comparison of these in single-phase and hydrolytic-acidogenic reactor.

## Discussion

SCOD, protein, and polysaccharide were detected to evaluate the effectiveness of sludge solubilization. VS reduction is an indication of sludge stability which is often used for assessing the effectiveness of a process in stabilizing sludge. The results showed that sludge disintegration and stabilization was improved by all pretreatments but their efficiency was varied. VS reduction was proportional to the increase in SCOD. Thermal pretreatment was the most efficient when compared with two other single treatment methods. In addition, ultrasonic-thermal pretreatment was only slightly improved over thermal treatment alone. In view of energy-saving and cost-reducing, thermal pretreatment was considered to be an appropriate method on anaerobic digestion of oil refinery waste activated sludge. A similar result from Bougrier *et al*.^13^ reported that thermal treatment was better than ultrasound treatment on activated sludge disintegration. Kim *et al*.^10^ also studied thermal (121 °C), chemical (7 g/L NaOH) and ultrasonic (42 kHz, 120 min) pretreatments on sludge prior to batch anaerobic digestion. It was realized that thermal pretreatment showed the best performance comparing with others. Besides, VFA concentration was less than 150 mg/L in all pretreated sludge samples (data not shown). Based on the calculation of 1 g VFA equal to 1.067 g COD, VFA only contributed to less than 6% SCOD. VFA concentration reflected the degree of acidification. It demonstrated that pretreatment was useful for sludge solubilization but had few effects on sludge acidification.

Temperature and pH are important parameters in anaerobic digestion. Temperature affects biochemical reactions in many ways, including reaction rate, reaction pathway, microorganism community, substrate utilization rate and the degree of acidification. In addition, acidogens can be functioned at a wide pH range between 5.0 and 8.5, while predominant acidogens and microbial community was far different resulting in different metabolic products^14^. VFA is easily lab measurable parameter which can give essential process information and is considered as effective parameter to evaluate the performance of hydrolytic-acidogenic stage^15^. It’s obviously that VFA production in thermolphilic hydrolytic-acidogenic process was much higher than that in mesophilic digestion. The high temperature not only improved the acidification rate but also shortened the reaction time. It could be an increase of diffusion coefficients at higher temperature contributed to better mass transfer of the system^16^. Similar report from Gavala *et al*.^17^ also illustrated that high temperature enhanced the hydrolytic-acidogenic process. In addition, weak-acidic condition was favorable in VFA production. Similar results were also occurred in anaerobic digestion of piggery wastewater^18^. Such condition also benefits for phase-separation, as methanogen are alkalescent dependent.

The composition of produced VFA was an important factor for anaerobic digestion, since methane production was affected by VFA concentration and composition. Different VFA components would lead to dissimilar methane production efficiencies. The results indicated that VFA composition was affected by HRT. The relative content of acetic and butyric was highest at HRT of 2 days. It is generally recognized that acetic and butyric acids were favorable substrates for methanogens, and a high level of propionic acid restrained the activity of methanogenic archaea^19^. Therefore, short HRT (2 days) was appropriate for hydrolytic-acidogenic stage. Moreover, it was reported that anaerobic digestion was inhibited when VFA concentration was above 6 g/L or propionic acid concentration was higher than 3.2 g/L^20^,^21^. In this study, the concentration of VFA and propionic acid never exceeded the inhibition levels during the whole experimental stages. Furthermore, the dominant VFA components would be favorable for the following methanogenic phase.

The comparative study of two-phase and single-phase anaerobic digestion demonstrated that two-phase anaerobic digestion had significant advantages in methane production and organic removal. Hydrolytic-acidogenic phase provided more soluble organics and VFA, which could be immediately used for methanogenesis. The enhanced VFA concentration not only promoted biogas production but also improved methane recovery ratio. In a word, hydrolytic-acidogenic stage facilitated methanogenic phase. In single-phase anaerobic digestion, the undesirable performance of organic removal and methane production could be explained by (1) organics from crude oil included refractory aromatics and heterocyclic compounds, (2) some organics were toxic to methanogen. The operating condition of single-phase system was not proper for thriving of acidogenic bacteria, especially the microbes with ability to degrade refractories to readily biodegradable VFAs. These refractory organics would be long-term existing in the reactor, thereby, inhibited the activity of methanogen. Therefore, two-phase anaerobic digestion facilitated the treatment of oil refinery waste activated sludge. It is confirmed that two-phase anaerobic digestion is beneficial in high-strength wastes treatment.

Two-phase anaerobic digestion has proved to be effective on oil conversion in this study. The oil content was 5.3% g/g-TS in the refinery waste activated sludge and up to 77.3% of oil was degraded. GC-MS results demonstrated that organic abundance and peak intensity in the initial sample (Fig. 4A) was much higher than that in the sludge residue (Fig. 4B) which further confirmed that most of oil compounds were removed. Oil components in the refinery waste activated sludge were mainly alkanes. After anaerobic biodegradation, the relative content of alkanes decreased seriously, which indicated that most of alkanes were biodegraded, such as dodecane, tetradecane, pentadecane, octadecane and 2,6,10-trimethylpentadecane. Goates *et al*.^22^ also found that alkanes were easily biodegradable substances. The relative abundance of esters in the sludge residue was much higher than that in the initial sample, indicated that esters were accumulated in the reactor. The peak intensity at 27.87 min, 28.87 min, and 33.96 min corresponding to dibutyl phthalate, 10,13-eicosadienoic acid methyl eater, and 1,2-benzenedicarboxylic acid diisooctyl ester were almost same in the two samples. It appears that these esters were not biodegraded by two-phase anaerobic digestion due to high structure stability (long side-chain) and poor water solubility. It was reported that the increasing length of ester side-chain would decrease the biodegradability of ester^23^. The relative content of alcohols increased from 7.35% to 19.38%. The increased alcohols could be metabolic products or intermediates of refractory substances.

Microbial community analysis provided crucial information to understand the anaerobic digestion process which may help to improve its efficiency. To reveal the microbial communities in anaerobic digestion of refinery waste activated sludge, high-throughput sequencing was conducted. The biodiversity results showed a less species richness and taxonomic diversity was occurred in the two-phase anaerobic digestion system. The detailed information at class and family levels demonstrated that methanogens acclimated and enriched in the phased methanogenic reactor. Low abundance of methanogens in the hydrolytic-acidogenic reactor was due to the short HRT and low pH condition, and the scarcity in single-phase reactor could be growth inhibition by toxic oil compounds. In addition, *Methanosaeta* and *Methanosarcina* which utilize acetate to produce methane were dominant in methanogenic reactor^24^. It is demonstrated that hydrolytic-acidogenic sludge with high VFA concentration (acetate as main VFA component) facilitated the growth of acetatetrophic methanogens in the methanogenic reactor.

Most bacterial genes were affiliated to Bacteroidetes, Firmicutes and Protebacteria phyla, which were common bacterial phyla in anaerobic digestion process associated with hydrolysis, acidogensis and acetogenesis^25^,^26^. Besides, the high abundance of *Caloramator, Ureibacillus, Dechloromonas, Petrobacter*, and *T78* revealed specific enrichment of certain bacterial groups in the hydrolytic-acidogenic reactor. The genus *Caloramator* include thermophilic anaerobes, and most species can degrade protein, carbohydrates and polysaccharide to acetate, ethanol, formate and hydrogen^27^,^28^. Members of *Ureibacillus* are known to possess hydrolytic function which was the major contributor to increase in soluble organics^29^. Notably, *Dechloromonas, Petrobacter* and *T78* were capable of anaerobic degradation of oil organics, including benzene compounds and oil hydrocarbons^30^,^31^. These enriched consortiums produced small organic molecules for methanogen and degraded toxic oil compounds. Therefore, the specific functional genera played important roles in anaerobic digestion of oil refinery waste activated sludge. In comparison, oil-decomposing bacteria were relatively low in the single-phase reactor. In methanogenic reactor, except predominant methanogen, abundance of syntrophic bacteria was also recognized. These bacteria included species in the family Porphyromonadaceae and the genus *Anaerobranca*. Bacteria in the family Porphyromonadaceae participated in fermentation of complex carbohydrates and proteins^32^, while, species in the genus *Anaerobranca* were capable of degrading VFA with 4–8 carbon atoms to acetate when co-cultured with methanogen^33^. These bacteria provided acetate for *Methanosaeta* and *Methanosarcina* to accelerate methane production and SCOD removal.

Selective proliferation of fast-growing microbes in hydrolytic-acidogenic and methanogenic reactors could be attributed to phase separation and optimized acidogenic operating conditions. The enrichment of functional bacteria was necessary for efficient anaerobic digestion of refinery sludge. The optimized two-phase anaerobic digestion helped to intensification of dominant population in each reactor, thereby good performance of two-phase anaerobic digestion of oil refinery waste activated sludge could be achieved, particularly with an advantage of resource recovery in comparison with single-phase anaerobic digestion.

## Materials and Methods

### Oil refinery waste activated sludge and inoculum sludge

Oil refinery waste activated sludge was collected from an oil refinery wastewater treatment plant in Yanshan petrochemical company (Beijing, China) with a capacity of processing crude oil 8.5 million tons per year. Samples were stored at 4 °C before use. The characteristics of the sludge were shown in Table 3. The seed sludge was obtained from a steady operation digester at a municipal wastewater treatment plant in Beijing, China. The pH, alkalinity, TS, and VS of the seed sludge were 7.5, 3050 mg CaCO_3_/L, 25.7 g/L and 16.3 g/L, respectively.

Prior to inoculation for hydrolytic-acidogenic reactor, an acclimation for transition from a methanogenic system to a hydro-acidogenic system was conducted. Seed sludge with the volume of 0.85 L was fermented at pH 6.0–6.5, and fed with refinery sludge progressively by reducing the sludge retention time from 15 to 3 days. HCl with the concentration of 0.1 mol/L was used to adjust pH in the reactor. The acclimated sludge was used for inoculation of hydrolytic-acidogenic reactor.

### Pretreatment experiments

A comparative study on thermal, alkali, ultrasonic and ultrasonic-thermal pretreatments was performed. For ultrasonic treatment, 200 mL sludge samples were ultrasonicated for 60 min by an ultrasonic homogenizer HC-S-40 (HuaChuang, China) operating at 20 kHz, 1 W/mL. The sample-containing beaker was submerged in an ice bath to minimize any temperature increase caused by ultrasonication. Alkali pretreatment was carried out in a 500 mL flask at room temperature (approximately 25 °C). NaOH solution with concentration of 6 mol/L was added into 200 mL sludge samples until a fixed pH to 12. Samples were mixed for 60 min using a magnetic stirrer at 200 rpm and then neutralized with HCl. A 300 mL hydrothermal Teflon reactor (Dongtai, China) was used for the thermal pretreatment. 200 mL sludge samples were added into the Teflon reactor and then was heated at 170 °C for 60 min in a muffle furnace. For the combination of ultrasonic-thermal pretreatments, 200 mL sludge was first ultrasonicated and then followed by thermal pretreatment with the same methods mentioned above. The selected treatment time, temperature, pH and other conditions of each method was pre-optimized. VS, SCOD, soluble protein and polysaccharide in each pretreated sludge samples were determined to evaluate the effectiveness of each pretreatment. The optimum pretreatment method was selected and the pretreated sludge was used in the following experiments.

### Optimization of hydrolytic-acidogenic phase

In order to optimize the hydrolytic-acidogenic conditions, a serial of experiments was conducted under different initial pHs (5.0, 5.5, 6.0, 6.5, and 7.0), temperatures (35 °C and 55 °C), and HRTs (1, 2, 3, 4, 5 days). The experiments were carried out in Fermentor bottles (SIBATA, Japan) with total volume of 1.0 L. In each reactor, the effective volume was 0.8 L and the ratio of inoculum to substrate was 1:4. The reactor was operated in semi-continuous mode. Pretreated sludge was fed to each reactor twice a day. To ensure steady-state condition, each HRT was kept running for at least three cycles for data collection. VFA was determined in each condition and the composition of VFA was also detected at the optimum condition.

### Comparison of two-phase and single-phase anaerobic digestion

**Figure 6:**
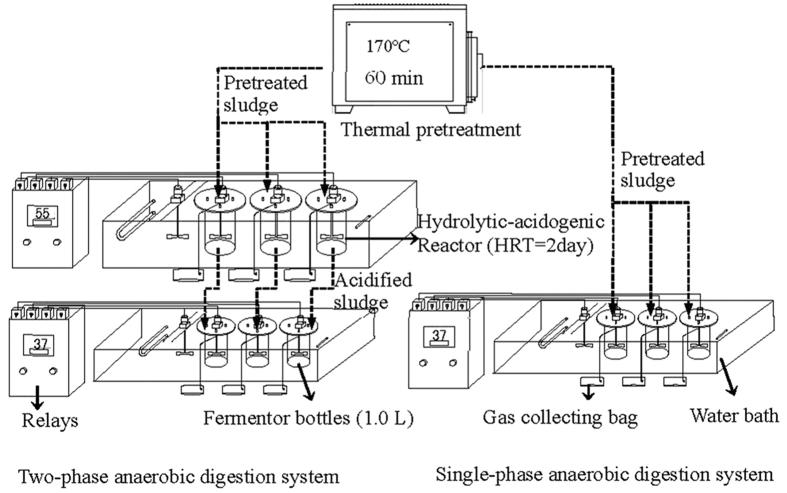
The schematics of two-phase anaerobic digestion system and single-phase anaerobic digestion system.

A comparative study of two-phase and single-phase anaerobic digestion was performed to evaluate organic removal and methane production, shown in Fig. 6. The experiments were carried out in batch Fermentor bottles (SIBATA, Japan) with total volume of 1.0 L. All of them were incubated in a water-bath reactor agitated at 180 rpm and 37 °C for mesophilic anaerobic digestion. Acidified sludge from the hydrolytic-acidogenic reactor was digested in the methanogenic reactor, and pretreated sludge was digested in the single-phase reactor. Both reactors were inoculated with 20% seed sludge from the municipal wastewater treatment plant and flushed with N_2_ for 5 min to replace air. Biogas was collected in gas collection bags (CYD-1L, XuXi Equipment, China), and the volume was daily recorded by an injection syringe (200 mL, ZhongChi Maker, China). Biogas composition, VFA and SCOD concentration were determined every day. To minimize random error, three replicates were performed in parallel and mean values were determined for each dataset.

### Microbial community structure analysis

The microorganism communities in single-phase reactor, hydrolytic-acidogenic reactor and methanogenic reactor were investigated by Illumina high-throughput sequencing. DNA from each sample was extracted with a PowerSoil DNA Isolation kit (Mobio Laboratories Inc., Carlsbad, USA) following the manufacturer’s instruction. The homogenization step was performed at 5000 rpm for 20 s by Precellys^®^ 24 (Bertin Technologies, France). DNA templates were stored at −20 °C before further analyses. The V3 and V4 regions of 16 S rDNA gene were selected for PCR, and two universal primers, 340 F (5′-TCCTACGGGAGGCAGCAGT-3′) and 805 R (5′-GGACTACCAGGGTATCTA ATCCTGTT-3′) were used for amplification. All PCR reactions were conducted in 50 μL solutions with 38.8 μL of ddH_2_O, 5 μL of 10 × Buffer A (Kapa Biosystem, USA), 1 μL 10 mM dNTPs, 2 μL primers, 0.2 μL of Kapa Taq DNA Polymerase (Kapa Biosystem, USA) and 1 μL DNA template. The procedure for PCR amplification was performed as follows: 95 °C for 3 min, followed by 30 cycles of denaturation at 95 °C for 30 s, annealing at 50 °C for 30 s, elongation at 72 °C for 60 s and a final extension at 72 °C for 7 min. After that, 5 μL PCR products were mixed with 5 μL 1X loading buffer (containing SYBR green), and electrophoresis was conducted on 1.5% agarose gel to detect positive amplified bands. Finally, PCR products were purified with a gel extraction kit (MinElute Gel Extraction Kit, Qiagen, Germany). Amplicons from different samples were pyrosequenced on an Illumina MiSeq platform at SinoGenoMax Co., Ltd (Beijing, China).

For quality control, the reads containing one or more ambiguous bases (“N”) were removed. Illumina sequencing generated a pair of reads from the two ends (paired-end reads) for one DNA fragment. Paired-end reads from the original DNA fragments were merged by SOAPdenovoto software to avoid overlaps. Then, the tag sequences were sorted into different individual files according to the barcodes of all samples. Sequence data were processed by read trimming and identification of V3–V4 sequences, followed by filtering and assigning the operational taxonomic units (OTUs). OTUs were identified with a cutoff of 97% identity. The reads from filtered OTUs were processed using the Quantitative Insights into Microbial Ecology (QIIME) program to construct a representative sequence which was selected to annotate taxonomic information. Besides, the species richness (Chao 1) and diversity indices (Shannon) of the microbial community were calculated by QIIME program.

### Analytical methods

COD, TS, VS, TN and VFA were determined according to the standard methods^34^. pH was measured daily by a pH meter (FE20, Mettler, Switzerland). Oil content in the oil refinery waste activated sludge was determined by an oil-content analyzer (GG330-OIL-420, ZhongXi, China) according to national standard method in China^35^. Oil extraction followed Soxhlet extraction method^36^ and carbon tetrachloride was used as extractant. The organic chemical composition of the extracted oil was characterized using a gas chromatograph-mass spectrometer (GC-MS). The GC-MS conditions followed the method of Wang *et al*.^31^. The sludge samples were centrifuged at 6000 rpm for 15 min and then filtered through a 0.45 μm disc-filter (JinTeng, China). Obtained supernatant was used to determine SCOD, VFA, protein and polysaccharide. Soluble protein was determined according to the Lowry method^37^, and polysaccharide was determined using the Anthrone-method^38^. The composition of VFA including acetic acid, propionate acid, butyric acid, and valeric acid was determined by a Gas chromatograph (SP-2100, Beijing Beifen-Ruili analytical Instrument Co. Ltd.) equipped with a FID detector and a capillary column. The temperatures of oven, injector and detector were 130 °C, 250 °C and 260 °C, respectively. Biogas composition was analyzed by this gas chromatograph equipped with a TCD detector and a Porapak-Q column. Helium gas was used as carrier gas, and temperatures of oven, injector and detector were 80 °C, 100 °C and 150 °C, respectively.

## Additional Information

**How to cite this article**: Wang, Q. *et al*. Potential and optimization of two-phase anaerobic digestion of oil refinery waste activated sludge and microbial community study. *Sci. Rep.* **6**, 38245; doi: 10.1038/srep38245 (2016).

**Publisher’s note:** Springer Nature remains neutral with regard to jurisdictional claims in published maps and institutional affiliations.

## Figures and Tables

**Table 1:** The effects of temperature and pH on VFA production at each experimental HRTs.

	VFA concentration (mg/L)				
	HRT = 1 day	HRT = 2 days	HRT = 3 days	HRT = 4 days	HRT = 5 days
Temperature (pH = 6.0)					
55 °C	1131.2 ± 51.0	1801.5 ± 44.4	1764.8 ± 42.2	1499.3 ± 15.5	1311.7 ± 22.3
35 °C	120.3 ± 13.3	120.5 ± 10.0	390.6 ± 12.7	486.4 ± 17.2	389.8 ± 9.8
pH (55 °C)					
5.0	672.4 ± 21.0	1132.5 ± 35.5	1178.5 ± 33.2	1052.0 ± 35.0	770.1 ± 41.4
5.5	1069.2 ± 55.1	1609.8 ± 50.0	1592.6 ± 33.5	1546.6 ± 24.6	1132.5 ± 22.4
6.0	1131.2 ± 27.5	1801.5 ± 47.6	1764.8 ± 41.3	1499.3 ± 36.6	1311.7 ± 36.0
6.5	1460.3 ± 21.7	2754.0 ± 25.5	2142.1 ± 26.5	1875.3 ± 30.0	1475.5 ± 33.3
7.0	1391.3 ± 36.6	2098.7 ± 35.5	1960.7 ± 43.6	1834.1 ± 44.8	1333.8 ± 25.9

95% confidence intervals are shown for each value.

**Table 2:** Relative abundance of methanogens and bacteria at family/genus level (>1% relative abundance) in the single-phase, hydrolytic-acidogenic and methanogenic reactors.

Phylum	Class	Genus/family	Single-phase	Aacidogenic phase	Methano-genic phase
Euryarchaeota	Methanomicrobia		8.1%	6.2%	17.6%
		*Methanoregula*	3.9%	2.7%	1.0%
		*Methanosaeta*	0.1%	0.3%	8.2%
		*Methanoculleus*	3.2%	1.7%	1.9%
		*Methanosarcina*	0.2%	0.2%	5.8%
		*Methanospirillum*	0.4%	1.1%	0.7%
Firmicutes	Clostridia		11.6%	23.7%	35.5%
		*Caloramator*	5.8%	15.1%	0.7%
		*Anaerobranca*	0.2%	0.2%	6.1%
	Bacilli		1.3%	7.5%	0.4%
		*Ureibacillus*	0.1%	5.3%	0.2%
		*Bacillus*	0.8%	1.8%	0.2%
	Erysipelotrichi	*RFN20*	0.6%	0.2%	2.1%
Proteobacteria	Betaproteobacteria		11.6%	17.7%	3.6%
		*Petrobacter*	0.3%	4.3%	1.8%
		*Dechloromonas*	1.6%	2.7%	1.0%
	Gammaproteo-bacteria		7.9%	4.9%	2.2%
		*Pseudomonadaceae*	2.7%	2.3%	0.2%
		*Halomonadaceae*	1.5%	0.1%	0.0%
Bacteridetes	Bacteroidia		20.6%	15.3%	27.4%
		*Porphyromonadaceae*	1.3%	0.8%	24.5%
		*Bacteroidaceae*	11.7%	10.0%	2.2%
Spirochaetes	Spirochaetes		3.1%	3.6%	0.3%
		*Treponema*	2.8%	3.3%	0.2%
Chloroflexi	Anaerolineae	*T78*	2.3%	4.5%	1.2%
others			32.9%	16.4%	9.7%

**Table 3:** Physical features of the refinery waste activated sludge.

pH	SCOD mg/L	Alkalinity g/L CaCO_3_	TS g/L	VS g/L	Soluble Carbohydrates mg/L	Soluble Proteins mg/L	Oil content (% g/g-TS)	TN mg/L	Ratio VS/TS (%)
6.9 ± 0.3	165.0 ± 6.5	1.700 ± 0.2	15.5 ± 0.5	11.5 ± 0.5	16.1 ± 1.0	45.2 ± 1.5	5.6 ± 0.3	158.1 ± 0.8	74.1 ± 0.5

95% confidence intervals are shown for each value.
